# Clinical and Histopathological Risk Factors for Multiple Cutaneous Squamous Cell Carcinomas

**DOI:** 10.1177/12034754261427420

**Published:** 2026-03-10

**Authors:** Marika Lounas, Tiina Luukkaala, Teea Salmi, Leea Ylitalo, Juha Jernman, Johanna Palve, Niina Korhonen

**Affiliations:** 1 Department of Dermatology, Tampere University Hospital, Wellbeing Services County of Pirkanmaa, Tampere, Finland; 2 Faculty of Medicine and Health Technology, Tampere University, Tampere, Finland; 3 Research, Development and Innovation Centre, Tampere University Hospital, Tampere, Finland; 4 Health Sciences, Faculty of Social Sciences, Tampere University, Tampere, Finland; 5 Department of Pathology, Tampere University and Fimlab Laboratories, Wellbeing Services County of Pirkanmaa, Tampere, Finland; 6 Department of Plastic Surgery, Tampere University Hospital, Wellbeing Services County of Pirkanmaa, Tampere, Finland

**Keywords:** cutaneous squamous cell carcinoma, keratinocyte carcinoma, nonmelanoma skin cancer, actinic keratosis, Bowen’s disease

## Abstract

**Background:**

Some patients with cutaneous squamous cell carcinoma (cSCC) may suffer from multiple tumors but little is known of the patient- or tumor-related factors associated with multiple cSCCs.

**Objective:**

To investigate the proportion of patients having more than 1 cSCC tumor and to explore whether there are factors that associate with multiple cSCC tumors.

**Methods:**

Histopathological diagnoses of “cSCC” from patients treated in Pirkanmaa, Finland, between 2006 and 2020, were obtained from the pathology database to identify patients with cSCC and to obtain the number of all diagnosed cSCC tumors. Clinical records were reviewed to collect information on the patients and their tumors.

**Results:**

A total of 1421 patients with 1988 cSCC tumors were identified. One-fifth of the patients (n = 285) had multiple cSCC tumors during the study period. A multivariable-adjusted model showed the patients with multiple cSCCs to be more often men and to have actinic keratosis, Bowen’s disease, and basal cell carcinomas (BCCs) more frequently than the patients with a single cSCC. The median time between the index and the second cSCC was 13 (interquartile range 2-34) months and the second tumor appeared at the same location as the index tumor in 71% of the cases. An invasion depth of more than 2 mm was associated with multiple tumors.

**Conclusions:**

Multiple tumors appeared in 20% of the patients with cSCC, and were more common among those with previous or simultaneous premalignant skin lesions and BCCs.

## Introduction

The incidence of cutaneous squamous cell carcinoma (cSCC) has been increasing worldwide placing a marked burden on health care systems.^1-3^ Even so, the true impact of these malignant tumors is often an underestimation, since some of the epidemiological studies have not to assessed cSCCs separately from other keratinocyte carcinomas, while others have counted only 1 tumor/person or 1 tumor/person/year.^4^ It has been suggested previously that the real number of cSCCs requiring treatment may increase by more than 30% if each tumor is considered individually rather than counting only 1 cSCC tumor/patient.^5^

Cumulative exposure to ultraviolet (UV) radiation is the most important causal factor for cSCC, although immunosuppression, smoking, certain β-human papilloma virus subtypes and treatment with BRAF inhibitor, thiazide diuretics, and high doses of psoralen and UVA therapy, for example, have also been associated with cSCC.^6,7^ Moreover, hematological malignancies^8^ and solid organ transplantations have been shown to increase the risk of cSCC tumors,^8,9^ and in some individuals, cSCCs may be related to certain genetic diseases or syndromes such as xeroderma pigmentosum or epidermodysplasia verruciformis.^10^ Previous studies focusing on multiple sSCCs are rare, but they do suggest that a history of actinic keratosis (AK)^11,12^ or other skin cancers [basal cell carcinoma (BCC) and/or malignant melanoma (MM)] might also be associated with multiple cSCCs.^12^ Contradictory information exists about the effects of gender, as some studies^8,13^ have indicated that men have more multiple tumors than women and others have not found a significant association.^12^ Overall, more information is needed on whether there are other patient or tumor-related risk factors that predispose individuals to multiple cSCCs. This would be of value, since cSCCs are a growing problem, especially in ageing populations, and limited resources could be focused better on prevention and treatment at an early stage.

There are no specific follow-up guidelines for patients with subsequent cSCC tumors, and the follow-up recommendations for patients with cSCC in general vary between different studies and guidelines.^14^ In the European guideline, for instance, a life-long follow-up is recommended for organ transplant recipients, and patients with leukemia or xeroderma pigmentosum, while for patients with some other form of immunosuppression and for immunocompetent patients the frequency and duration of the follow-up is based on features of the cSCC tumor itself. It has been established, however, that patients with multiple cSCCs have a higher risk of local recurrence and nodal disease, so that optimal follow-up protocols would be especially important for them.^15^

We have reported previously on the incidence and characteristics of cSCCs in a defined cohort in Finland between 2006 and 2020.^1^ This current study is aimed at determining the proportion of patients with multiple cSCCs in the cohort and identifying those characteristics of both the tumors (including histological details) and the patients that may be associated with the development of multiple tumors.

## Materials and Methods

As described in our previous publication,^1^ histopathological diagnoses of “cSCC” were obtained from Fimlab’s pathology database between January 1, 2006, and December 31, 2020. Fimlab is a provider of laboratory services in the Pirkanmaa region of Finland. Only invasive cSCCs were included, that is, in situ cSCC, nonprimary cSCC, and samples with an uncertain diagnosis were excluded. The clinical records of Tampere University Hospital, which is the region’s largest hospital and the tertiary referral center for Pirkanmaa, were reviewed for all the cSCC patients identified.

If the patient had multiple primary cSCC tumors, each was regarded as an independent tumor. Data on the tumors were gathered individually, considering the histological information obtained from biopsies and excisions for each tumor, and information from the clinical records. Details of histopathological features, including degrees of differentiation and invasion depths, were collected for each tumor. The tumors were also assigned to one of the following 8 anatomical locations based on the clinical records: lip, eyelid, ear, face, neck/scalp, trunk, upper extremity, or lower extremity. Tumors of the head were pooled for these analyses, and tumors in the anogenital area and oral cavity were excluded altogether.

Registered demographic factors included the patient’s age at the diagnosis of the index tumor (the first tumor to occur during the study period 2006-2020) and sex, and data on immunosuppression were also collected and classified into the following types: solid organ transplantation, lymphoma, leukemia, rheumatoid arthritis, treatment with BRAF inhibitor, and HIV infection. Finally, information on previous or simultaneous AK, Bowen’s disease, BCC, and MM was gathered from the clinical records. The institutional review board of Tampere University Hospital, Finland, approved this retrospective study.

### Statistical Methods

The clinical data and demographic variables for the cSCCs were evaluated using descriptive statistics and frequency tabulations. Despite the skewed distribution, the numbers of tumors were expressed using means and standard deviations, as these provide more information on the number of tumors than do medians and ranges, where only the maximum value differs from 1 (tumor). Multivariable-adjusted risk factors for more than 1 tumor were searched for using logistic regression and the results shown as odds ratios with 95% confidence intervals. If multiple tumors were detected, the data from the index tumor were modeled. Statistical analyses were performed using IBM SPSS Statistics for Windows, version 26.0 software (IBM Corp, Armonk, NY, USA). Two-sided *P* values under .05 were considered statistically significant.

## Results

A total of 1421 patients with 1988 cSCC tumors were identified, their median age at the time of the index cSCC tumor diagnosis being 81 years (interquartile range 74-86) years, and 54% of them were men (Supplemental Table 1). The number of individual tumors per patient varied from 1 to 28, with 80% of the patients (1136 out of 1421) having a single cSCC during the study period, whereas 20% (285 out of 1421) had 2 or more cSCC tumors (Supplemental Figure 1) and accounted for 43% of the total number of tumors (852/1988). Thus, the inclusion of multiple cSCCs per patient, increased the total number of cSCC tumors by 29% compared to the recording of only 1 cSCC per patient.

Altogether, 148 patients (10%) were immunosuppressed, the most common reason for this being solid organ transplantation (n = 56, 38%). Other frequent reasons for immunosuppression were rheumatoid arthritis (n = 41, 28%), lymphoma (n = 24, 16%), and leukemia (n = 23, 16%). Where 9% of the patients with a single cSCC were immunosuppressed, the figure among those with multiple cSCCs was 18% (Supplemental Table 1).

Out of the patients with a single cSCC tumor, 62% had AK, 40% Bowen’s disease, and 34% BCC, whereas the respective figures for patients with multiple cSCCs were 86%, 67%, and 54%. The proportion of well-differentiated index tumors was similar among the patients with single (48%) and multiple (47%) tumors (Supplemental Table 1).

The multivariable-adjusted model showed male sex, immunosuppression, AK, Bowen’s disease, BCC, and tumor invasion depth over 2 mm to be associated with multiple tumors (Supplemental Table1), but it should be remembered that almost 40% of the invasion depth data were missing.

According to this adjusted model, multiple tumors were more likely to appear if the index tumor was located on the upper extremity rather than the head (Supplemental Table 1). Otherwise, the tumor locations did not differ noticeably between the patients with single or multiple cSCCs. Meanwhile, among the patients with multiple tumors, 71% of the second tumors appeared in the same location as the index tumor (Supplemental Table 2), and 85% of those whose index tumor was located in the head area had a second tumor in the same area.

The median time between the index tumor and the second tumor during this period in the patients with multiple tumors was 13 (interquartile range 2-34) months, and the time to the appearance of a new tumor began to shorten from the fourth tumor onwards (Supplemental Figure 2).

## Discussion

One-fifth of the patients diagnosed with cSCC were found to have developed 2 or more cSCC tumors during the 15 year period studied here, and when all their subsequent cSCC tumors were taken into account, ~29% more cSCC tumors were reported than when considering only each patient’s first tumor. This is important information when discussing the epidemiological impact of multiple tumors. In fact, even higher figures of 30% to 50% more tumors have been reported previously when counting each individual tumor.^5^ Our results are not necessarily generalizable to all other populations; however, as the incidence rates of cSCCs in Australia and New Zealand, for example, are much higher (175-467/100 000 person-years)^13^ than in northern latitudes (15-77/100 000 person-years).^6^ The lesion-based incidence of cSCC has not been well investigated, but it has been estimated to vary between 330 and 1125/100 000.^13^ Nonetheless, the risk of subsequent tumors appears to increase as the number of tumors increases, so that patients with non-first cSCC have been reported to have an over 50% risk of acquiring a new tumor within 2 years and a more than 70% risk after 5 years.^4^ These findings suggest that it is important not to underestimate the real burden of cSCCs on the health care system and on the patients affected in this way.

Since immunosuppression is a recognized patient-related risk factor for subsequent cSCC tumors,^9,12^ we reviewed the types of immunosuppression separately and found that solid organ transplantation had the strongest association with multiple tumors. However, multiple cSCC tumors are not only a problem for immunosuppressed patients, as more than 75% patients who had a cSCC without known immunosuppression have been reported to be at risk of developing a subsequent tumor.^16^ In our cohort, the proportion of immunocompetent patients among those with multiple cSCCs was 83%. It has been reported previously that men have more multiple cSCCs than women,^5,8,9,12,17^ and our findings support this conclusion. It is plausible that after being diagnosed with skin cancer, for example, melanoma, women examine their skin for abnormal markings and protect it more carefully than do men.^18^

The patients with multiple cSCC tumors had BCC and premalignant lesions (AK and Bowen’s disease) more frequently than did the patients with a single cSCC tumor. A history of previous keratinocyte cancer seems to be a predictor for subsequent BCC and SCC development, but there is limited information focusing on cSCCs without combining it with BCCs.^19-21^ However, Moseley et al have previously reported that prior AK is associated with multiple cSCCs, and also found that a history of (melanoma or nonmelanoma) skin cancer has a similar association.^12^ It was not possible for us to obtain a timeline for other skin cancers in relation to cSCCs, but it was established that prior or current AK was associated with multiple cSCCs.

When considering tumor-related risk-factors for multiple tumors, we could not detect any differences in tumor differentiation between the patients with single and multiple cSCCs. Previous studies contain conflicting information as to whether the degree of differentiation is associated with the development of multiple tumors.^12,22^ Rodriguez et al found that a poor tumor grade was a risk factor for subsequent tumors as compared to well, moderate, or unknown grades,^22^ whereas Moseley et al did not find any association between tumor differentiation and subsequent cSCCs.^12^ These contradictory results suggest a need for further research in larger populations. Our study suggests however, for the first time, that index tumor invasion depths of over 2 mm could be associated with multiple tumors. However, since information on the depth of invasion was missing in almost 40% of cases, the result is only indicative and the matter requires further investigation.

The median time between the index tumor and the second tumor, and also the second and third tumors, was 13 to 14 months. Eggermont et al obtained similar results, as the time between the first and second tumors in their study was 1.4 years and that between the second and third tumors 1.2 years.^8^ Since the interval between the occurrence of tumors varied from 2 to 22 months, it is difficult to recommend an optimal schedule for follow-up, but at any rate, patients with multiple cSCCs require a follow-up, as the risk of subsequent tumors has been reported to rise as the number of previous cSCCs increases.^8^

The location of the second cSCC tumor in the patients with multiple tumors was the same as that of the index tumor in ~70% of cases. Ciążyńska et al, studying the location of subsequent keratinocyte skin cancers, found that the primary tumor and subsequent tumors tended to occur in the same anatomical location, but cSCCs and BCCs were not observed separately in their study.^23^ In our cohort, there was an indication that multiple tumors occurred more frequently in patients with an index tumor in the upper limb than in those with an index tumor in the head. All in all, tumors were commonly located in areas exposed to the sun, emphasizing the role of UV radiation in their pathogenesis. We found some variability in the subsequent tumor locations, especially in anatomical locations other than the head. These findings emphasize that it is of utmost importance in clinical practise to perform a total-body skin examination, not only examine the site of previously diagnosed tumors, due to the possible presence of other skin conditions or skin cancers.^6^

As we have found before, the incidence of cSCCs is increasing,^1^ and it is also evident in the light of the present work that multiple cSCC tumors are a significant concern. To reduce the number and incidence of cSCCs in the future, the focus should be on preventative health care measures. Primary prevention should focus mainly on avoiding UV radiation,^24^ in connection with which behavioral interventions such as the regular use of a sunscreen have been reported to be effective in reducing the incidence of AK and cSCC.^6^ Secondary prevention of cSCCs is also important and should be focused mainly on the treatment of precursor lesions.^25^ Little is known, however, of the effect of treatment for AK in preventing cSCC. At least patients with multiple lesions and/or extensive diffuse AK/field cancerization may benefit from prophylactic treatment.^6,26^ Chemoprevention with nicotinamide or oral retinoids may be considered in patients with a high risk of developing aggressive cSCC or multiple tumors.^6^

The notable strengths of this study were its long follow-up time and its possession of data on all the cSCC tumors reported in a geographically well-defined area. Furthermore, the clinical records were reviewed to ensure that the tumors in question were true separate primary tumors and were collected as precisely as possible in a retrospective study with data on tumor locations and histological details such as invasion depth and degree of differentiation. However, due to the retrospective nature of our study it also had some limitations. Almost 40% of the invasion depth data was missing, and it was not possible to collect all the relevant information on each cSCC and patient. Data on tumor macroscopic sizes were only collected from 2016 onwards, for example, and even then, they were reported accurately enough for inclusion in the study only in a small proportion of cases. Furthermore, as the study included cSCC tumors diagnosed during a defined period, it could not be confirmed that the index tumor was in fact the patient’s first. The data also represent a specific geographical area in Northern Europe so that the results cannot necessarily be generalized to other areas.

## Conclusion

Using a well-defined cohort and reliable registers demonstrated that as many as 1 in 5 patients with cSCC developed at least 1 subsequent cSCC tumor during a 15 year period. Multiple cSCCs seemed to be more common among men and were further associated with immunosuppression and other skin cancers and premalignant skin lesions. Patients with a tumor in the head area are especially prone to developing subsequent tumors in the same area. However, despite having an opportunity to analyze the histological details of the tumors, we did not find any marked tumor-related risk factors that were associated with multiple tumors. The significance of the depth of invasion and of location of the tumor on an upper extremity should be further studied. As risk stratifications in guidelines are based mostly on cSCC tumor details, this poses a clinical challenge concerning the patient follow-up plans. Nonetheless, as multiple cSCCs, along with other types of skin cancer, are a growing public health issue, both primary and secondary prevention should be considered.

## Supplemental Material

sj-docx-1-cms-10.1177_12034754261427420 – Supplemental material for Clinical and Histopathological Risk Factors for Multiple Cutaneous Squamous Cell Carcinomas Supplemental material, sj-docx-1-cms-10.1177_12034754261427420 for Clinical and Histopathological Risk Factors for Multiple Cutaneous Squamous Cell Carcinomas by Marika Lounas, Tiina Luukkaala, Teea Salmi, Leea Ylitalo, Juha Jernman, Johanna Palve and Niina Korhonen in Journal of Cutaneous Medicine and Surgery

sj-docx-2-cms-10.1177_12034754261427420 – Supplemental material for Clinical and Histopathological Risk Factors for Multiple Cutaneous Squamous Cell Carcinomas Supplemental material, sj-docx-2-cms-10.1177_12034754261427420 for Clinical and Histopathological Risk Factors for Multiple Cutaneous Squamous Cell Carcinomas by Marika Lounas, Tiina Luukkaala, Teea Salmi, Leea Ylitalo, Juha Jernman, Johanna Palve and Niina Korhonen in Journal of Cutaneous Medicine and Surgery

sj-tif-3-cms-10.1177_12034754261427420 – Supplemental material for Clinical and Histopathological Risk Factors for Multiple Cutaneous Squamous Cell Carcinomas Supplemental material, sj-tif-3-cms-10.1177_12034754261427420 for Clinical and Histopathological Risk Factors for Multiple Cutaneous Squamous Cell Carcinomas by Marika Lounas, Tiina Luukkaala, Teea Salmi, Leea Ylitalo, Juha Jernman, Johanna Palve and Niina Korhonen in Journal of Cutaneous Medicine and Surgery

sj-tif-4-cms-10.1177_12034754261427420 – Supplemental material for Clinical and Histopathological Risk Factors for Multiple Cutaneous Squamous Cell Carcinomas Supplemental material, sj-tif-4-cms-10.1177_12034754261427420 for Clinical and Histopathological Risk Factors for Multiple Cutaneous Squamous Cell Carcinomas by Marika Lounas, Tiina Luukkaala, Teea Salmi, Leea Ylitalo, Juha Jernman, Johanna Palve and Niina Korhonen in Journal of Cutaneous Medicine and Surgery
