# Supplementary material for: Clinical and Histopathological Risk Factors for Multiple Cutaneous Squamous Cell Carcinomas
Source: J Cutan Med Surg. 2026 Mar 10;30(4):363–8. doi: 10.1177/12034754261427420 (PMC13478435; doi:10.1177/12034754261427420)
Supplement: Supplementary file 1 [file sj-docx-1-cms-10.1177_12034754261427420.docx]

**Table 1**. Characteristics of patients with cutaneous squamous cell carcinoma (N=1,421) at the time of the index tumour (since 2006), and risk factors for multiple tumours^a^.

|  |  |  |  |  | Multiple vs. single tumours | | | | | |
| --- | --- | --- | --- | --- | --- | --- | --- | --- | --- | --- |
|  | Patients (N=1,421) | Tumours  (N=1988) | | | Single  (n=1136) | | Multiple  (n=285) | | Multivariable adjusted | |
|  | N | N | Mean | (Sd) | n | (%) | n | (%) | OR | (95% CI) |
| **Sex** |  |  |  |  |  |  |  |  |  |  |
| Men | 766 | 1155 | 1.51 | (1.69) | 589 | (52) | 177 | (62) | 1.40 | (1.04-1.87) |
| Women | 655 | 833 | 1.27 | (0.84) | 547 | (48) | 108 | (38) | 1.00 |  |
| **Age years** |  |  |  |  |  |  |  |  |  |  |
| <75 | 376 | 592 | 1.57 | (2.17) | 301 | (26) | 75 | (26) | 1.00 |  |
| 75-79 | 248 | 381 | 1.54 | (1.34) | 186 | (16) | 62 | (22) | 1.17 | (0.77-1.78) |
| 80-84 | 308 | 405 | 1.31 | (0.80) | 247 | (22) | 61 | (21) | 0.83 | (0.54-1.26) |
| ≥85 | 489 | 610 | 1.25 | (0.66) | 402 | (35) | 87 | (30) | 0.74 | (0.50-1.10) |
| **Immunosuppression** |  |  |  |  |  |  |  |  |  |  |
| No | 1273 | 1669 | 1.31 | (0.90) | 1038 | (91) | 235 | (83) | 1.00 |  |
| Yes | 148 | 319 | 2.16 | (3.22) | 98 | (9) | 50 | (17) | 1.89 | (1.25-2.84) |
| **AK_~~*~~_** |  |  |  |  |  |  |  |  |  |  |
| No | 471 | 519 | 1.10 | (0.36) | 431 | (38) | 40 | (14) | 1.00 |  |
| Yes | 950 | 1469 | 1.55 | (1.63) | 705 | (62) | 245 | (86) | 2.72 | (1.84-4.04) |
| **Bowen** |  |  |  |  |  |  |  |  |  |  |
| No | 773 | 909 | 1.18 | (0.63) | 678 | (60) | 95 | (33) | 1.00 |  |
| Yes | 648 | 1079 | 1.67 | (1.87) | 458 | (40) | 190 | (67) | 2.09 | (1.53-2.85) |
| **BBC** |  |  |  |  |  |  |  |  |  |  |
| No | 878 | 1095 | 1.25 | (0.87) | 746 | (66) | 132 | (46) | 1.00 |  |
| Yes | 543 | 893 | 1.64 | (1.89) | 390 | (34) | 153 | (54) | 1.43 | (1.06-1.91) |
| **MM** |  |  |  |  |  |  |  |  |  |  |
| No | 1333 | 1843 | 1.38 | (1.34) | 1072 | (94) | 261 | (92) | 1.00 |  |
| Yes | 88 | 145 | 1.65 | (1.64) | 64 | (6) | 24 | (8) | 1.03 | (0.61-1.74) |
| **Invasion (mm)** |  |  |  |  |  |  |  |  |  |  |
| <1 | 66 | 91 | 1.38 | (1.44) | 56 | (5) | 10 | (3) | 1.00 |  |
| 1.0-2.0 | 418 | 577 | 1.38 | (1.15) | 339 | (30) | 79 | (28) | 1.44 | (0.69-3.03) |
| >2.0 | 397 | 569 | 1.43 | (1.66) | 309 | (27) | 88 | (31) | 2.21 | (1.05-4.65) |
| Unknown | 540 | 751 | 1.39 | (1.27) | 432 | (38) | 108 | (38) | 1.83 | (0.88-3.80) |
| **Differentation** |  |  |  |  |  |  |  |  |  |  |
| Well | 674 | 938 | 1.39 | (1.27) | 540 | (48) | 134 | (47) | 1.00 |  |
| Moderate to poor | 680 | 941 | 1.38 | (1.43) | 549 | (48) | 131 | (46) | 0.89 | (0.66-1.19) |
| Unknown | 67 | 109 | 1.63 | (1.57) | 47 | (4) | 20 | (7) | 1.54 | (0.84-2.82) |
| **Location** |  |  |  |  |  |  |  |  |  |  |
| Head | 1088 | 1466 | 1.35 | (1.08) | 879 | (77) | 209 | (73) | 1.00 |  |
| Trunk | 101 | 160 | 1.58 | (2.83) | 84 | (7) | 17 | (6) | 0.77 | (0.43-1.37) |
| Upper limb | 144 | 243 | 1.69 | (1.88) | 102 | (9) | 42 | (15) | 1.57 | (1.02-2.40) |
| Lower limb | 88 | 119 | 1.35 | (0.84) | 71 | (6) | 17 | (6) | 1.49 | (0.81-2.72) |
|  |  |  |  |  |  |  |  |  |  |  |

Abbreviations: AK= Actinic keratosis; Bowen = Bowen’s disease; BCC= Basal cell carcinoma; MM= Malignant melanoma; N=total number of participants; n=number of participants in a subset; OR=Odds Ratio; CI=Confidence interval

^a^The effects on later multiple tumours were modelled using logistic regression analysis and the results were expressed as OR with 95% CI. The status of each risk factor was derived from the patient’s index (first) tumour during 2006-2020.
