# Supplementary material for: Clinical and Histopathological Risk Factors for Multiple Cutaneous Squamous Cell Carcinomas
Source: J Cutan Med Surg. 2026 Mar 10;30(4):363–8. doi: 10.1177/12034754261427420 (PMC13478435; doi:10.1177/12034754261427420)
Supplement: Supplementary file 2 [file sj-docx-2-cms-10.1177_12034754261427420.docx]

Table 2. Proportion of second tumours occurring in the same location as the index tumour in patients with multiple tumours (n=285) between 2006 and 2020

|  | | Index tumour | | Second tumour in the same location as the index tumour | |
| --- | --- | --- | --- | --- | --- |
| Location | | n | (%) | n | (%; 95% CI) |
| **Head** | | 209 | (73) | 178 | (85; 80-90) |
|  | Lip and ear | 41 | (14) | 10 | (24; 11-38) |
|  | Other locations on the face, eyelid, scalp, neck | 168 | (59) | 91 | (54; 47-62) |
| **Trunk** | | 17 | (6) | 5 | (29; 8-51) |
| **Upper extremity** | | 42 | (15) | 11 | (26; 13-39) |
| **Lower extremity** | | 17 | (6) | 9 | (53; 29-77) |
| **Total** | | **285** | **(100)** | **203** | **(71; 65-77)** |
